# A Novel CCR5 Mutation Common in Sooty Mangabeys Reveals SIVsmm Infection of CCR5-Null Natural Hosts and Efficient Alternative Coreceptor Use *In Vivo*

**DOI:** 10.1371/journal.ppat.1001064

**Published:** 2010-08-26

**Authors:** Nadeene E. Riddick, Emilia A. Hermann, Lamorris M. Loftin, Sarah T. Elliott, Winston C. Wey, Barbara Cervasi, Jessica Taaffe, Jessica C. Engram, Bing Li, James G. Else, Yingying Li, Beatrice H. Hahn, Cynthia A. Derdeyn, Donald L. Sodora, Cristian Apetrei, Mirko Paiardini, Guido Silvestri, Ronald G. Collman

**Affiliations:** 1 Department of Medicine, University of Pennsylvania School of Medicine, Philadelphia, Pennsylvania, United States of America; 2 Department of Microbiology, University of Pennsylvania School of Medicine, Philadelphia, Pennsylvania, United States of America; 3 Department of Pathology and Laboratory Medicine, University of Pennsylvania School of Medicine, Philadelphia, Pennsylvania, United States of America; 4 Yerkes National Primate Research Center, Emory University, Atlanta, Georgia, United States of America; 5 Departments of Medicine and Microbiology, University of Alabama at Birmingham, Birmingham, Alabama, United States of America; 6 Department of Pathology and Laboratory Medicine, Emory University, Atlanta, Georgia, United States of America; 7 Seattle Biomedical Research Institute, Seattle, Washington, United States of America; 8 Department of Microbiology and Molecular Genetics, University of Pittsburgh Center for Vaccine Research, Pittsburgh, Pennsylvania, United States of America; Fred Hutchinson Cancer Research Center, United States of America

## Abstract

In contrast to HIV infection in humans and SIV in macaques, SIV infection of natural hosts including sooty mangabeys (SM) is non-pathogenic despite robust virus replication. We identified a novel SM CCR5 allele containing a two base pair deletion (Δ2) encoding a truncated molecule that is not expressed on the cell surface and does not support SIV entry *in vitro*. The allele was present at a 26% frequency in a large SM colony, along with 3% for a CCR5Δ24 deletion allele that also abrogates surface expression. Overall, 8% of animals were homozygous for defective CCR5 alleles and 41% were heterozygous. The mutant allele was also present in wild SM in West Africa. CD8+ and CD4+ T cells displayed a gradient of CCR5 expression across genotype groups, which was highly significant for CD8+ cells. Remarkably, the prevalence of natural SIVsmm infection was not significantly different in animals lacking functional CCR5 compared to heterozygous and homozygous wild-type animals. Furthermore, animals lacking functional CCR5 had robust plasma viral loads, which were only modestly lower than wild-type animals. SIVsmm primary isolates infected both homozygous mutant and wild-type PBMC in a CCR5-independent manner *in vitro*, and Envs from both CCR5-null and wild-type infected animals used CXCR6, GPR15 and GPR1 in addition to CCR5 in transfected cells. These data clearly indicate that SIVsmm relies on CCR5-independent entry pathways in SM that are homozygous for defective CCR5 alleles and, while the extent of alternative coreceptor use in SM with CCR5 wild type alleles is uncertain, strongly suggest that SIVsmm tropism and host cell targeting *in vivo* is defined by the distribution and use of alternative entry pathways in addition to CCR5. SIVsmm entry through alternative pathways *in vivo* raises the possibility of novel CCR5-negative target cells that may be more expendable than CCR5+ cells and enable the virus to replicate efficiently without causing disease in the face of extremely restricted CCR5 expression seen in SM and several other natural host species.

## Author Summary

SIV causes AIDS in macaques, like HIV-1 does in humans, but not in its natural host species such as sooty mangabeys (SM). It is therefore important to understand infection in natural hosts, including the mechanisms and cellular targets of infection. SIV replication in SM is thought to exclusively use CCR5 as its entry coreceptor, which mediates viral entry in conjunction with CD4 and is the main determinant of target cell tropism. However, other molecules also function as SIV coreceptors *in vitro*. We discovered that inactivating mutations in the CCR5 gene are common among SM and, furthermore, homozygous mutant animals lacking functional CCR5 still become infected and have high viral loads. *Ex vivo*, SM lymphocytes can be infected independently of CCR5, and several alternative entry coreceptors are used by SIV from both CCR5+ and CCR5-null animals. Thus, SIV infection in SM is mediated by other coreceptors in addition to CCR5, suggesting that these molecules together may determine tropism and cell targeting *in vivo*. These results provide new insight into an important model of nonpathogenic natural host infection, and identify a novel role for alternative entry pathways suggesting a potentially broader range of target cells *in vivo* than currently recognized.

## Introduction

HIV-1 emergence into the human population resulted from cross-species transmission of SIVcpz from chimpanzees (*Pan troglodytes*), which itself resulted from transmission and subsequent recombination of SIVs infecting primates on which chimpanzees prey [1], [2]. Similarly, both simian AIDS caused by SIVmac/smm in rhesus macaques (RM; *Macaca mulatta*) and HIV-2 infection of humans originated from cross-species transmission of SIVsmm from naturally infected sooty mangabeys (SM; *Cercocebus atys*) [3], [4], [5]. In marked contrast to pathogenic infections leading to AIDS in non-natural hosts, infection in natural host species including SM is typically non-progressive [6], [7], [8]. Importantly, the benign nature of SM infection *in vivo* is not due to overall restricted viral replication, as both nonpathogenic natural host and pathogenic nonnatural host infections are characterized by robust viremia [9], [10], [11], [12]. This observation indicates that immunodeficiency virus replication and pathogenesis are not inextricably linked. Thus, understanding natural host infection has become a high priority for identifying key features of infection *in vivo* that regulate pathogenesis and, potentially, identify opportunities to modulate disease apart from or in addition to suppressing overall virus replication through pharmacologic or immune mechanisms.

HIV and SIV entry into target cells is initiated by binding of the viral envelope glycoprotein (Env) to cell surface CD4, followed by structural changes that enable interactions with a seven transmembrane G protein coupled cell surface receptor that then triggers fusion. HIV-1 isolates use CCR5 or CXCR4 or both, and *in vitro* use other molecules infrequently. The restricted expression of CCR5 mainly on memory CD4+ T cells, but broader expression of CXCR4 on both memory and naïve subsets, is thought in part to underlie the accelerated disease progression seen in individuals in whom CXCR4-using HIV-1 variants emerge late in the course of infection [13], [14], [15], [16]. In contrast, SIV strains use CCR5 almost universally and very rarely use CXCR4. Sooty mangabeys express very low levels of CCR5 on their CD4+ T cells, a mechanism by which replication might be regulated *in vivo* and restrict transmission and pathogenesis [17], [18]. However, most strains of SIV use a number of other alternative coreceptors in *in vitro* assays, such as CXCR6 (STRL33), the orphan receptors GPR1 and GPR15, and several others [19], [20], [21]. Despite the efficient use of such alternative entry pathways by SIVmac and SIVsmm isolates in transfected cells, infection and cell targeting *in vivo* is generally thought to be dependent on CCR5 [22]. Notably, however, the proportion of CD4+ T cells depleted and/or infected at a given time in macaques and mangabeys may exceed the proportion of cells with detectable CCR5 expression, raising the possibility that other pathways in addition to CCR5 might be utilized [23], [24].

Although both natural and non-natural host infections result in high level virus replication, several distinguishing features provide clues as to possible causes for the distinct outcomes. It is long believed that in addition to CD4+ T cell destruction, pathogenesis involves an inability to effectively replenish these populations [25], [26]. In pathogenic rhesus macaque infection, damage to the CD4+ T central memory (Tcm) subpopulation appears to play a central role in the inability of infected animals to replenish CD4+ T effector and effector memory (Tem) cells depleted by infection [27]. It has been recently found that cell-associated viral loads in CD4+ Tcm are considerably lower in SM than RM, despite equivalent or higher Tem infection levels, which might enable better immune cell homeostasis in infected SM (G.S. and M.P; unpublished observations). Another difference is the presence of chronic generalized immune activation in infected humans and RM, whereas natural hosts display generalized immune activation during acute infection that then rapidly resolves [11], [28], [29], [30]. Chronic generalized immune activation may contribute to accelerated T cell turnover and ultimate depletion, and is believed to result in large part from translocation of gut microbial products due to gastrointestinal barrier damage during acute infection [31]. However, vigorous virus replication and extensive CD4+ T cell depletion in gut mucosal tissue occur in both natural and non-natural hosts [24], [32], [33], [34]. A potentially important difference is Th17 CD4+ T cells, which play a critical role in defense against bacteria at mucosal sites, and are lost in HIV-1 and SIVmac rhesus macaque infection but spared in infected natural hosts [35], [36], [37], [38]. Thus, the factors regulating CD4+ T cell subset targeting *in vivo* may be central to defining the outcome of infection in natural or non-natural hosts.

Interestingly, evolution appears to have favored mutations in the CCR5 gene that abrogate surface expression of this molecule. An allele containing a 32 base pair frameshift deletion in human CCR5 that abrogates cell surface expression (CCR5Δ32) is present at a frequency of 10% in the Caucasian population, resulting in about 18% heterozygous and 1% homozygous individuals. CD4+ cells from individuals homozygous for CCR5Δ32 are resistant to infection by CCR5-using HIV-1 isolates *in vitro* but permissive for strains that can use CXCR4, and the essential role for CCR5 in HIV-1 transmission and infection is demonstrated by the finding that individuals homozygous for CCR5Δ32 are highly resistant to infection [39], [40]. Furthermore, heterozygous individuals can be infected but show lower viral loads and slower disease progression, in association with lower levels of CCR5 expression [41], [42]. Red-capped mangabeys (RCM; *Cercocebus torquatus*) are the natural host of SIVrcm, and a 24 base pair in-frame deletion (CCR5Δ24) that also abrogates surface expression is present at an allelic frequency of 87% [43]. As a result, ≥70% of RCM are homozygous for the mutation and do not express CCR5, and the one known exception to exclusive CCR5 dependence by SIV *in vivo* is SIVrcm, which uses CCR2b for entry and cannot use CCR5. The same CCR5Δ24 mutant allele was also reported in two different populations of SM, which are closely related to RCM, but with a low allelic frequency of 4% and no animals homozygous for the allele were found [43], [44].

In this study, we identified a novel 2 base pair deletion and frameshift mutation in SM CCR5 (CCR5Δ2) that results in a lack of surface expression and coreceptor function. The mutation is present at a 26% allele frequency in the large Yerkes National Primate Research Center (YNPRC) SM colony, and together with the previously-described Δ24 allele results in 8% of animals lacking functional CCR5. However CCR5-null SM are susceptible to natural and experimental SIVsmm infection and exhibit robust viral replication. This is the first clear evidence for *in vivo* alternative coreceptor use by SIVsmm in its natural hosts, which provides an explanation for the efficient use of alternative coreceptors by the SIVsmm/mac family of viruses. This data also suggests that cell targeting and tropism in sooty mangabeys is linked to expression and use of both CCR5 and additional alternative entry pathways, and identifies a third example of convergent evolution resulting in nonfunctional mutant CCR5 alleles among primate species.

## Results

### Identification of a novel mutation in the sooty mangabey CCR5 gene

**Figure 1:**
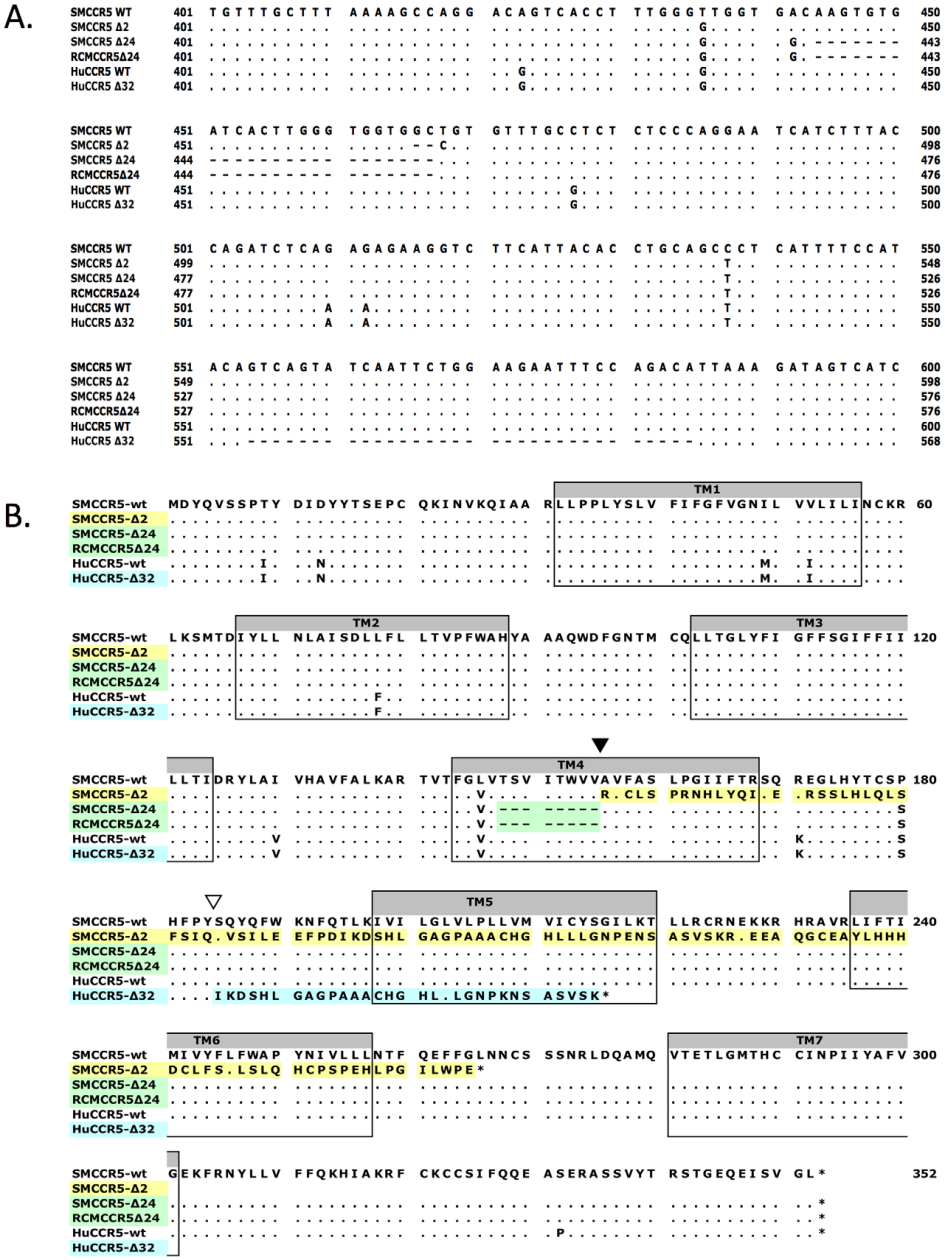
Sequence alignment of wild-type and mutant CCR5 genes. (A) Partial nucleotide sequence alignment and (B) predicted protein sequence alignment of the wild-type smCCR5 and smCCR5Δ2 alleles (GenBank HM246693 and HM246694), the CCR5Δ24 alleles previously described in sooty and red capped mangabeys (GenBank AF07473 and AAC62474), and human wild-type CCR5 and CCR5Δ32 alleles (GenBank DQ217934 and U66285). Residues identical to the wild-type smCCR5 molecule are indicated by ‘.’ and gaps are shown as dashes. Location of the frameshift resulting from the smCCR5Δ2 deletion in TM4 is indicated by an inverted black triangle while the huCCR5Δ32 frameshift is indicated by an inverted white triangle. Transmembrane domains are indicated by shaded boxes and mis-sense amino acid sequences resulting from mutations are highlighted.

We amplified the CCR5 coding sequence from SM genomic DNA, cloned it into an expression vector and analyzed several clones by sequence analysis. Cloning from four independent PCR reactions amplifying smCCR5 from one animal (FVq) resulted in the identification of two distinct alleles in each of the PCR amplifications (Figure 1). One was a wild-type allele, similar to published smCCR5 sequences (smCCR5 wt), and the other was a novel allele containing a two base pair deletion at nucleotides 466 and 467 of the coding sequence (smCCR5Δ2), which corresponds to the fourth transmembrane domain (TM4) of the smCCR5 protein (Figure 1A). This deletion causes a frameshift that results in a predicted protein with 110 missense amino acids prior to termination at residue 265 (Figure 1B). In addition to this deletion, the smCCR5Δ2 allele contains two nucleotide substitutions compared with the wild-type allele identified. The first is a 436T>G substitution resulting in a L146V amino acid change, which is also found in several published wild-type smCCR5 sequences. The second nucleotide substitution is 538C>T, but is masked in CCR5Δ2 due to the frameshift.

In addition to premature truncation of the protein, the mutation results in several charged amino acids predicted within TM4, as well as loss of both disulfide bonds (between the N-terminus and third extracellular loop (ECL), and between the first and second ECLs, respectively) that maintain secondary structure. These features suggest that the mutant protein is unlikely to be configured in a manner to allow proper membrane placement and either normal signaling or SIV/HIV entry coreceptor function.

There are several other mutations in primate CCR5 genes, including the well-studied 32 base pair frameshift mutation in human CCR5 (CCR5Δ32) [39], [40], [42] and a 24 base pair deletion (CCR5Δ24) that is common in RCM and also reported at low frequency in SM [43], [44]. Therefore, we examined the relationship between this SM CCR5Δ2 deletion and the Δ24 RCM/SM and human Δ32 deletions (Figure 1A & B). The smCCR5Δ2 deletion occurs in the same region of TM4, and overlaps the Δ24 deletion, which is characterized by multiple G-T-G repeats. In contrast, the human CCR5Δ32 deletion occurs approximately 90 bases downstream of smCCR5Δ2, within the second extracellular loop of the protein. Interestingly, the SM and RCM Δ24 alleles also contain the 436T>G and 538C>T substitutions seen smCCR5Δ2, which result in amino acids identical to those in human CCR5 at those respective sites (valine at position 146 and serine at position 180; Figure 1B).

Because the CCR5Δ24 mutation was previously reported to be present in animals at YNPRC [44], animals were screened for its presence by PCR and several Δ24 carriers were identified (Figure S1). We therefore generated a clone of the CCR5Δ24 coding region by PCR of genomic DNA from one heterozygous animal. Sequence analysis of this CCR5Δ24 clone was similar to the sequence previously described [43], [44], except for a non-coding 1026G>T substitution.

### Cell surface expression of mutant smCCR5Δ2

Previous studies have shown that proteins encoded by the mutant human CCR5Δ32 and RCM/SM CCR5Δ24 are not expressed on the cell surface [40], [44]. To test whether the smCCR5Δ2 mutant gene gave rise to a protein expressed on the cell surface, we transfected 293T cells with wild-type and mutant SM CCR5 expression plasmids, along with human CCR5, and measured surface expression by flow cytometry. Staining utilized mAb 3A9, which cross-reacts with both human and SM CCR5 and, importantly, recognizes an epitope in the N-terminus that is upstream of the mutation and should be detected if the predicted protein were expressed.

**Figure 2:**
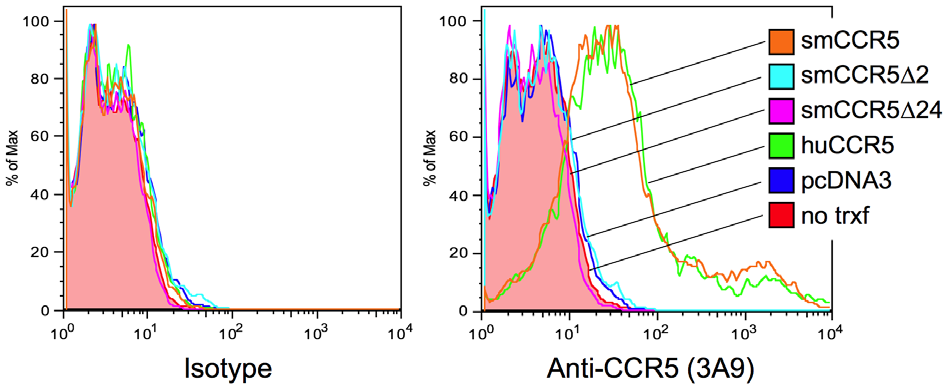
Surface expression of wild-type and mutant CCR5 variants *in vitro*. 293T cells were transfected with expression plasmids encoding smCCR5 wild-type (orange), smCCR5Δ2 (light blue), smCCR5Δ24 (magenta) and huCCR5-wt (green), along with empty expression plasmid (dark blue). Untransfected 293T cells (shaded red) serve as controls. Cells were stained with an anti-CCR5 mAb (clone 3A9; APC-conjugated), which recognizes the N-terminal region of both human and sooty mangabey CCR5 (right panel), or an isotype-matched control antibody (left panel).

As shown in Figure 2, surface expression of CCR5 was readily detected on cells transfected with SM or human wild-type CCR5. In contrast, neither the Δ2 nor Δ24 SM mutant CCR5 alleles gave rise to detectable surface expression. We have so far been unable to assess intracellular expression because of high nonspecific intracellular staining with 3A9 and other anti-N-terminal CCR5 antibodies in all cells tested so far (data not shown). From this data, we conclude that the frameshift mutation in smCCR5Δ2 results in a truncated protein that is not expressed on the cell surface. In addition, our observation that the CCR5Δ24 mutant allele is not expressed at the cell surface is consistent with previous reports that this deletion abrogates cell surface expression even though it is not a frameshift [43], [44]. We also asked if the mutant protein might have a dominant negative effect on wild-type CCR5 expression, but found no change in CCR5 staining if the wild-type CCR5 plasmid was co-transfected along with the Δ2 or Δ24 alleles (Figure S2).

### Prevalence of the smCCR5Δ2 allele in the YNPRC sooty mangabey population

Next we determined the prevalence of the smCCR5Δ2 allele in SM housed at the Yerkes National Primate Research Center (YNPRC), which is the largest captive colony of SM in the world (n = 202). All animals were initially screened using a discriminatory PCR assay that specifically identifies the smCCR5 wild-type and Δ2 alleles (Figure S1A). Animals were also screened for the Δ24 allele using primers that discriminate Δ24 from Δ2 and wild-type alleles based on amplicon size (Figure S1B). Results were then verified by direct bulk sequencing of genomic DNA amplicons that confirmed the presence or absence of homozygous genotypes, or demonstrated frameshifting with sequence overlap in heterozygous animals (Figure S1C).

The result from this analysis is shown in Table 1. Five of six possible genotypes were identified: 50.5% of the SM carried two wild-type CCR5 alleles; 37.6% were heterozygous for wild-type and Δ2 alleles, and 4% of the SM were heterozygous for the wild-type and Δ24 alleles. Notably, 6.4% of the SM were homozygous for the smCCR5Δ2 allele, and 1.5% carried both Δ2 and Δ24 mutant alleles (Table 1). Thus, nearly 8% of animals carry two CCR5 mutant alleles encoding defective CCR5 proteins. We did not identify any SM that were homozygous for the Δ24 allele.

**Table 1:** Genotypic frequencies in Sooty Mangabeys at YNPRC.

Genotype:	n	Observed frequency (%)^1^	Predicted frequency (%)^2^^,^ 3
W/W	102	50.5	50.8
W/Δ2	76	37.6	37.1
W/Δ24	8	4.0	3.9
Δ2/Δ2	13	6.4	6.8
Δ2/Δ24	3	1.5	1.4
Δ24/Δ24	0	0	0.1
Total	202		

1 Genotype distribution among all sooty mangabeys was determined by PCR and direct genomic sequence analysis.

2 Predicted genotype distribution was calculated by Punnett square analysis, based on allelic frequencies (W = 0.71; Δ2 = 0.26; Δ24 = 0.03) derived from observed genotypes.

3 Observed and predicted genotype frequencies are not significantly different (Chi-square test).

In this SM population, analysis of allelic frequencies showed 26% of alleles carried the novel Δ2 deletion, 3% carried the Δ24 deletion, and 71% were wild-type. Of note, the 3% frequency we found for the Δ24 allele is similar to the 4% allelic frequency described 12 years ago among SM housed at YNPRC [44]. We then used the allelic frequencies to calculate a predicted genotype distribution (Table 1 and Table S1). There was close agreement between predicted and observed genotypes (p = ns; Chi-square test), suggesting that the CCR5 alleles are in equilibrium in this population (of which ∼60% are SIV-infected), without evidence of selective pressure favoring or disfavoring any of the genotypes.

### Prevalence of mutant CCR5 alleles in other captive and wild African sooty mangabey populations

The YNPRC colony is the largest population of SM in the US and the close match between predicted and observed CCR5 genotype distributions suggested an absence of selective pressure for or against any specific genotype. However, we wished to determine the frequency with which these alleles and genotypes were present in other SM populations, so we analyzed genomic DNA obtained from 29 animals housed at the Tulane National Primate Research Center (TNPRC). Of note, many of the monkeys that founded the TNPRC colony originally came from YNPRC in the 1980s, but have been housed and bred separately since then. In this smaller population the Δ2 allele was present at a frequency of 19% and the Δ24 allele had a frequency of 5%. As shown in Table 2 and Table S2, the observed genotype frequencies in this population also do not differ from those predicted by allele frequencies.

**Table 2:** Observed and predicted genotype frequencies in Sooty Mangabeys housed at the TNPRC^1^.

Genotype:	n	Observed frequency (%)^2^	Predicted frequency (%)^3^^,^ 4
W/W	18	0.621	0.576
W/Δ2^5^	7	0.241	0.288
W/Δ24	1	0.034	0.078
Δ2/Δ2	1	0.034	0.036
Δ2/Δ24	2	0.069	0.020
Δ24/Δ24	0	0.000	0.003
Total	29		

1 All animals except one are SIV-infected.

2 Genotype distribution among all sooty mangabeys was determined by PCR and direct genomic sequence analysis.

3 Predicted genotype distribution was calculated by Punnett square analysis, based on allelic frequencies (W = 0.76; Δ2 = 0.19; Δ24 = 0.05) derived from observed genotypes.

4 Observed and predicted genotype frequencies are not significantly different (Chi-square test).

5 Includes one SIV-negative animal.

We also asked whether the CCR5Δ2 allele was present in SM in Africa. For this analysis we amplified CCR5 genes from fecal-derived host DNA samples from 33 wild-living animals in the Tai forest of Cote d'Ivoire [45]. Five animals carried both the CCR5Δ2 and wild-type alleles, and in one animal only the Δ2 allele was detected. An additional 2 animals carried both Δ24 and wild-type alleles, while the remaining animals revealed only wild-type sequences. Because this analysis utilized fecal DNA containing limiting quantities of host DNA, we can only be certain that both alleles were captured if animals were found to be heterozygous. Thus, while it is not possible to determine a precise allele frequency, these genotypes suggest a minimum allele frequency of 9% for CCR5Δ2 and 3% for CCR5Δ24 in this population. This result indicates that the Δ2 allele is also present in wild-living SM in Cote d'Ivoire, although likely at a lower frequency than in captive animals at YNPRC.

### Expression of CCR5 on SM CD4+ and CD8+ T cells *ex vivo*

**Figure 3:**
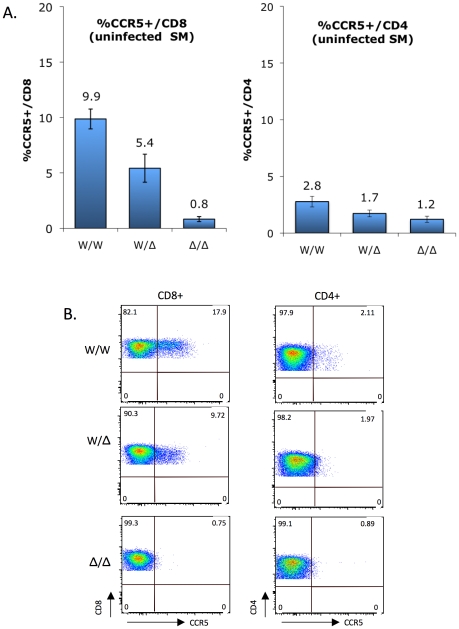
CCR5 surface expression on sooty mangabey CD4+ and CD8+ T cells *ex vivo*. (A) CCR5 staining on CD8+ T cells (left) and CD4+ T cells (right) of uninfected sooty mangabeys was carried out between 2004 and 2009, and analyzed according to genotype groups (mean ± SEM): CCR5 wild-type (W/W; n = 38), heterozygous (W/Δ; n = 34) and homozygous mutant (Δ/Δ; n = 7). Because both Δ2 and Δ24 alleles are functionally null, they were combined for this analysis. CCR5 expression by CD8+ T cells differs significantly between genotype groups (p<0.0001 by Kruskal-Wallis test), while the trend for CD4+ T cells does not reach statistical significance (p = ns). (B) FACS plots showing CCR5 staining on CD4+ and CD8+ T cells from representative homozygous wild-type, heterozygous and homozygous mutant animals.

Since overexpression studies in transfected cells suggested that neither this common smCCR5Δ2 nor the less common Δ24 proteins are expressed on the cell surface, we examined the relationship between genotypes and CCR5 expression on primary SM CD4+ and CD8+ T cells. To address this point, we analyzed CCR5 expression data that was available from animals housed at the YNPRC collected during periodic surveys between 2004 and 2009, focusing on uninfected animals, and grouped individuals according to their CCR5 genotypes. Since neither Δ2 nor Δ24 CCR5 alleles express following transfection, the two mutations were combined for the purposes of this analysis into a homozygous wild-type, heterozygous, and homozygous deletion allele groups (Figure 3).

As previously reported [18] and as shown in Figure 3, CCR5 expression is markedly greater on SM CD8+ T cells than CD4+ T cells. In both populations there was a gradation in the percentage of cells staining positive for CCR5 that correlated with CCR5 genotype status. The difference was particularly evident for CD8+ T cells, and the percentage of CCR5+/CD8+ T cells was 9.9%, 5.4% and 0.8% for wild-type, heterozygous and homozygous mutant groups, respectively (Figure 3A; p<0.0001 by Kruskal-Wallis test). This finding indicates that the CCR5Δ2 genotype acts as a determinant of CCR5 expression on CD8+ T cells. There was also a strong linear relationship between wild-type gene dosage and CCR5 expression for CD8 cells (R^2^ = 0.99996; p = 0.009 by Pearson's 2-tailed correlation coefficient), consistent with the absence of a dominant negative effect by the mutant alleles in transfected cells (Figure S2). A trend was also evident for CCR5 staining on CD4+ T cells among the three groups (2.8%, 1.7% and 1.2% in the wild-type, heterozygous and homozygous mutant groups, respectively), which did not reach statistical significance (p = ns by Kruskal-Wallis test) but is consistent with the notion that, although overall very low, CCR5 expression on CD4+ T cells is also regulated by CCR5 genotype.

Representative FACS plots showing CCR5 expression on CD4+ and CD8+ T cells from a homozygous wild-type, heterozygous and homozygous mutant animal are shown in Figure 3B. We think the low level (∼1%) of cells within the CCR5 gate for homozygous mutant animals likely represents background staining, rather than low levels of N-terminal expression given the result of transfection studies (Figure 2). Unfortunately, antibodies available that are directed at other epitopes in human CCR5 do not recognize SM CCR5.

### CCR5-null genotype does not protect sooty mangabeys from SIVsmm infection *in vivo*

In humans, the CCR5Δ32 homozygous genotype provides powerful protection against HIV-1 infection [39], [40], [42]. Therefore, we asked if animals homozygous for CCR5-null alleles were infected by SIVsmm. In order to more properly assess natural susceptibility, we restricted this analysis to YNPRC animals that were naturally infected (n = 120) and those with documented SIV-negative status (n = 72), and excluded animals in the colony known to have been experimentally infected (n = 10).

As shown in Table 3, we found that among SM with the CCR5 homozygous wild-type genotype, 65% were naturally infected while 35% were uninfected, and 62% of CCR5 heterozygous animals were infected and 38% were uninfected. Unexpectedly, among animals homozygous for CCR5-null alleles (n = 14), 50% were naturally infected and 50% were seronegative. Thus, animals lacking functional CCR5 genes are susceptible to natural SIVsmm infection. The slightly lower prevalence of SIV infection among animals in the CCR5-null group was not statistically significant (p = ns; Chi-square test). We also compared the distribution of genotypes within the SIV-negative and naturally-infected SIV populations (Table 4). Similarly, there were no significant differences in genotype distribution between the SIV serostatus groups (p = ns for each genotype group; 2-sample proportions test), although a slightly lower proportion of homozygous CCR5 mutant animals was seen in the infected animals compared with SIV-negative animals (5.8% vs. 9.7%; p = ns). Therefore, the CCR5-null genotype does not prevent natural acquisition of SIVsmm infection. Furthermore, neither heterozygosity nor homozygous null genotype appears to significantly influence SM susceptibility to SIVsmm infection *in vivo*, and while we cannot absolutely rule out a small effect, any protection that might be afforded by CCR5-null status would be slight. This result stands in marked contrast to the profound protective effect of CCR5Δ32 homozygosity in humans.

**Table 3:** Prevalence of naturally-acquired SIV infection among YNPRC sooty mangabeys based on CCR5 genotype.

Genotype^1^	SIV positive^2^ (n)	SIV negative^3^ (n)	% SIV infected^4^
W/W	63	34	65
W/Δ	50	31	62
Δ/Δ	7	7	50
Total	120	72	63

1 CCR5Δ2 and Δ24 alleles were grouped together as defective for expression (Δ).

2 Includes animals known to be infected naturally in the wild or in captivity, and excludes 10 animals infected experimentally.

3 Includes animals known to be SIV-negative at the time of last survey.

4 SIV prevalence does not differ significantly among the genotype groups (Chi-square test).

**Table 4:** Genotypic distribution among YNPRC sooty mangabeys based on SIV infection status.

Genotype^1^	SIV positive SM (n = 120)^2^^,^ 4	SIV negative SM (n = 72)^3^^,^ 4
W/W	0.525	0.472
W/Δ	0.417	0.431
Δ/Δ	0.058	0.097

1 CCR5Δ2 and Δ24 alleles were grouped together as defective for expression (Δ).

2 Includes animals known to be infected naturally in the wild or in captivity, and excludes 10 animals infected experimentally.

3 Includes animals known to be SIV-negative at the time of last survey.

4 Differences in genotype distribution among animals based on SIV infection status are not statistically significant (2-sample proportions test).

We also determined the genotypes of 10 animals at YNPRC that were infected experimentally. Five of these animals were homozygous for the CCR5 wild-type gene, three were heterozygotes (all W/Δ2) and two were homozygous for smCCR5Δ2. Furthermore, all but one of the TNPRC animals studied are SIVsmm-infected, including a mix of both natural infections and experimental inoculation done in earlier decades. Among the infected animals were three homozygous CCR5-null animals, while the one uninfected animal was heterozygous (W/Δ2). Therefore, SM naturally deficient in CCR5 expression are susceptible to experimental as well as natural SIVsmm infection, confirming that non-CCR5 entry pathways can mediate SM natural host infection.

### Influence of smCCR5 genotype on plasma viral loads and CD4 counts in SIVsmm-infected SM

**Figure 4:**
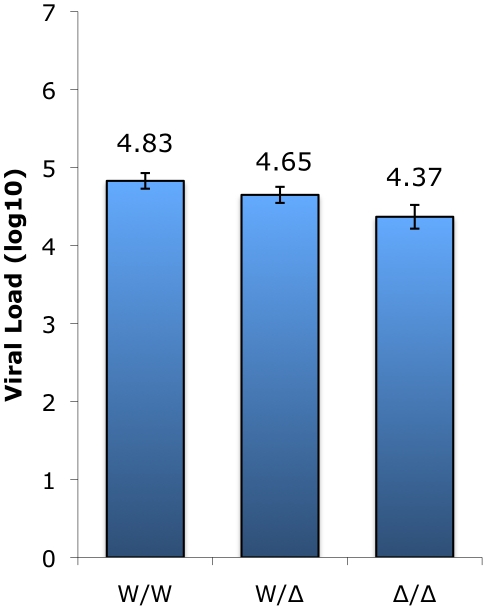
SIV plasma viral load measurements in infected sooty mangabeys between genotype groups. Plasma viral load measurements (VL log_10_; means ± SEM) collected in surveys carried out between 2004 and 2009 of infected animals in the wild-type (n = 60), heterozygous (n = 49), and homozygous mutant (n = 7) genotype groups. The Δ2 and Δ24 alleles were combined for this analysis. The difference in VL between homozygous wild-type and CCR5-null genotype groups (W/W vs. Δ/Δ) is statistically significant (p<0.05; Dunn's multiple comparison test) whereas the differences for the heterozygous group (W/Δ vs. W/W; W/Δ vs. Δ/Δ) does not reach statistical significance (p = ns; Dunn's multiple comparison test).

We next asked if CCR5 genotype affected plasma viral loads in infected animals. Based on previously collected data, the log_10_ viral load (mean ± SEM) was calculated for animals in each genotype group; for animals with multiple data points available, their mean viral load (log_10_) was used (Figure 4). This analysis showed robust viral loads in all genotype groups, with a modest but statistically significant gradient in VL dependent on the presence of a wild-type CCR5 allele (p = 0.005 by Kruskal-Wallis test). Animals possessing two wild-type alleles exhibited the highest viral load (4.83±0.10 log_10_), heterozygotes showed an intermediate level (4.65±0.10 log_10_), and infected animals with the CCR5-null genotype had the lowest VL (4.37±0.15 log_10_). Thus, there is approximately 0.5 log_10_ difference in viral load in animals with two wild-type compared with two CCR5-null alleles. The two important points here are that homozygous mutant animals have vigorous viral replication despite lacking functional CCR5, and simultaneously exhibit a small but significant difference in plasma viral load associated with increasing CCR5 gene dosage.

CD4 counts remain stable during chronic SIVsmm infection in the vast majority of animals, but some exceptions have been noted [46], [47], so we next asked if SIV-infected animals exhibit differences in CD4 counts depending on their CCR5 genotype. However, there was no significant difference in CD4+ T cell levels among the genotype groups, whether assessed based on absolute counts (Figure S3A; p = ns, Kruskal-Wallis) or on the basis of CD4 percentage (Figure S3B; p = ns, ANOVA). These results suggest that CD4+ T cell levels are maintained similarly in SIV-infected SM regardless of CCR5 genotype.

### smCCR5Δ2 does not support SIV entry *in vitro*

We then asked if there was any chance that the smCCR5Δ2 mutant allele, when expressed along with CD4, could support SIV infection *in vitro*. We considered it unlikely but thought it was necessary to test directly given the staining patterns by CCR5 mAb 3A9 in Δ2 homozygous primary mononuclear cells (Figure 3). We also considered it important to test using SIVsmm from a CCR5Δ2 homozygous infected animal, in case viral adaptation might have enabled use of an N-terminal region alone. Therefore, we generated pseudotype virions carrying Env glycoproteins cloned directly from plasma virus of an SIVsmm-infected CCR5Δ2 homozygous animal (FNp), along with pseudotypes carrying Envs cloned from plasma of an infected wild-type animal (FFv). Use of plasma virus ensured that these Envs were derived from actively replicating virus. Target 293T cells were co-transfected with CD4 plus plasmids encoding wild-type smCCR5, smCCR5Δ2, human CCR5 or an empty vector as a control, then infected with the primary SIVsmm pseudotypes. Of note, pseudotype virions carrying the FNp 5.1 Env were considerably less infectious than the other Envs and very large amounts of this virus were required to achieve equivalent infectious inocula, which generally resulted in high levels of background for this Env.

**Figure 5:**
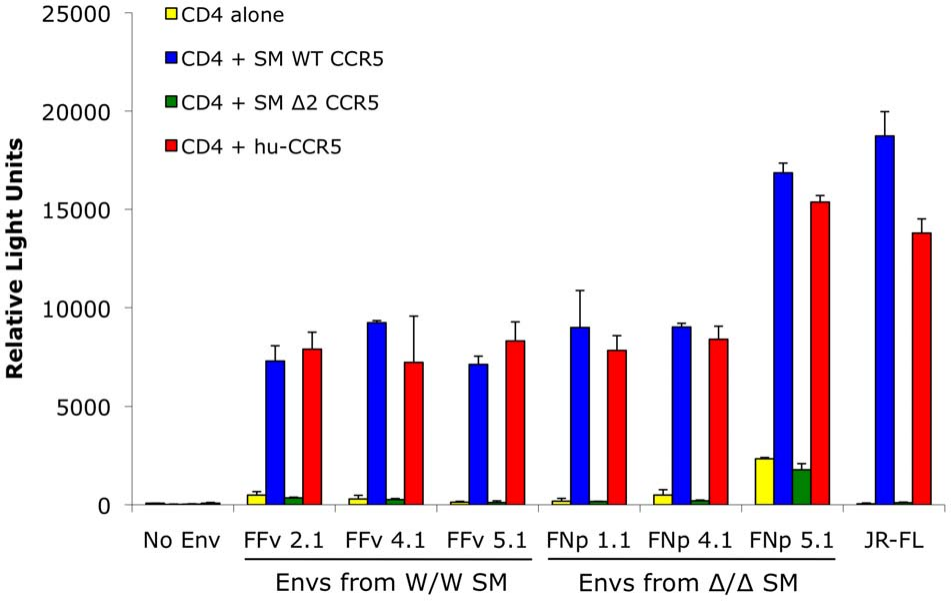
Mutant smCCR5Δ2 does not support SIV infection *in vitro*. 293T cells were transfected with CD4 alone (yellow bars) or in combination with wild-type smCCR5 (blue bars), smCCR5Δ2 (green bars) or wild-type human CCR5 (red bars). Target cells were infected with luciferase-expressing pseudotype virions carrying Env glycoproteins that were cloned from plasma of two SIVsmm-infected sooty mangabeys (FFv: W/W SM; FNp: Δ2/Δ2 SM). Pseudotypes carrying the R5-tropic HIV-1 Env JRFL and virions lacking envelope glycoproteins served as controls. Infection was measured by relative light units (RLU) in cell lysates 3 days after infection (mean ± SD).

As shown in Figure 5, wild-type smCCR5 and human CCR5 support infection of all SIVsmm variants to similar levels. In contrast, the smCCR5Δ2 allele does not support infection by any of the viruses. Importantly, CCR5Δ2 does not function as a coreceptor for Env variants from CCR5-null animals. Thus, SIVsmm infections in CCR5-null animals are mediated through pathways independent of CCR5.

### Use of alternative coreceptors by SIVsmm isolates

The data presented here indicates that SIVsmm must be able to use entry pathways other than CCR5 for replication *in vivo*. It is long known that many SIV strains use a number of alternative coreceptors in addition to CCR5 *in vitro*, although these viruses rarely use CXCR4. We therefore tested the ability of SIVsmm envelopes to mediate infection through human CCR2b, CCR3, CCR8, GPR1, GPR15 (BOB), CXCR6 and CXCR4. This analysis employed the uncultured SIVsmm envelope glycoproteins cloned from plasma of the CCR5-null (FNp) and homozygous wild-type (FFv) infected animals.

**Figure 6:**
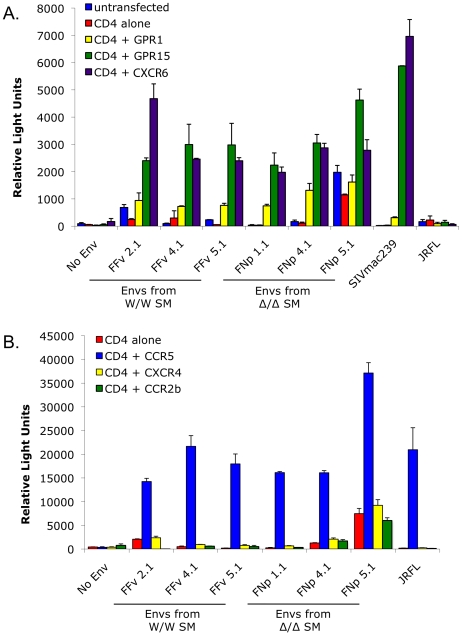
Alternative coreceptor utilization by SIVsmm Envs *in vitro*. 293T cells were transfected with CD4 alone or in combination with (A) the alternative coreceptors GPR1, GPR15 and CXCR6, or (B) CXCR4, CCR2b or CCR5. Target cells were then infected with luciferase-expressing pseudotype virions containing SIVsmm Envs from infected CCR5 wild-type (FFv: W/W SM) and CCR5-null (FNp: Δ2/Δ2 SM) animals. Envs from SIVmac239, HIV-1 JRFL and virions lacking Env served as controls. Infection was assayed by RLU (mean ± SD) measured 3 days after infection.

As shown in Figure 6, all SIVsmm envelope glycoproteins tested mediated entry into cells expressing CD4 in conjunction with GPR15 and CXCR6, while GPR1 was also used but less efficiently. In contrast, none of the SIVsmm envelope glycoproteins could use CXCR4 as a coreceptor, nor CCR3 or CCR8 (data not shown). Interestingly, SIVsmm envelope glycoproteins failed to use CCR2b, an entry pathway employed by SIVrcm in RCM that typically lack CCR5 due to a high prevalence of the Δ24 mutation [43]. Furthermore, the patterns of alternative coreceptor use were similar for envelope glycoproteins derived from CCR5-null and CCR5 wild-type animals, indicating that alternative coreceptor utilization is a feature shared by SIVsmm regardless of whether the host animal expresses CCR5. While absolute luciferase production varied among experiments, GPR15 and CXCR6 typically supported levels of infection similar to that mediated by CCR5 (Figure S4), although transfected targets likely represent maximum levels of potential utilization relative to primary cells that would express these molecules at physiological levels.

### SIVsmm infects sooty mangabey CCR5-null primary PBMC *in vitro* and infection of wild-type PBMC is not blocked by the CCR5 antagonist maraviroc

**Figure 7:**
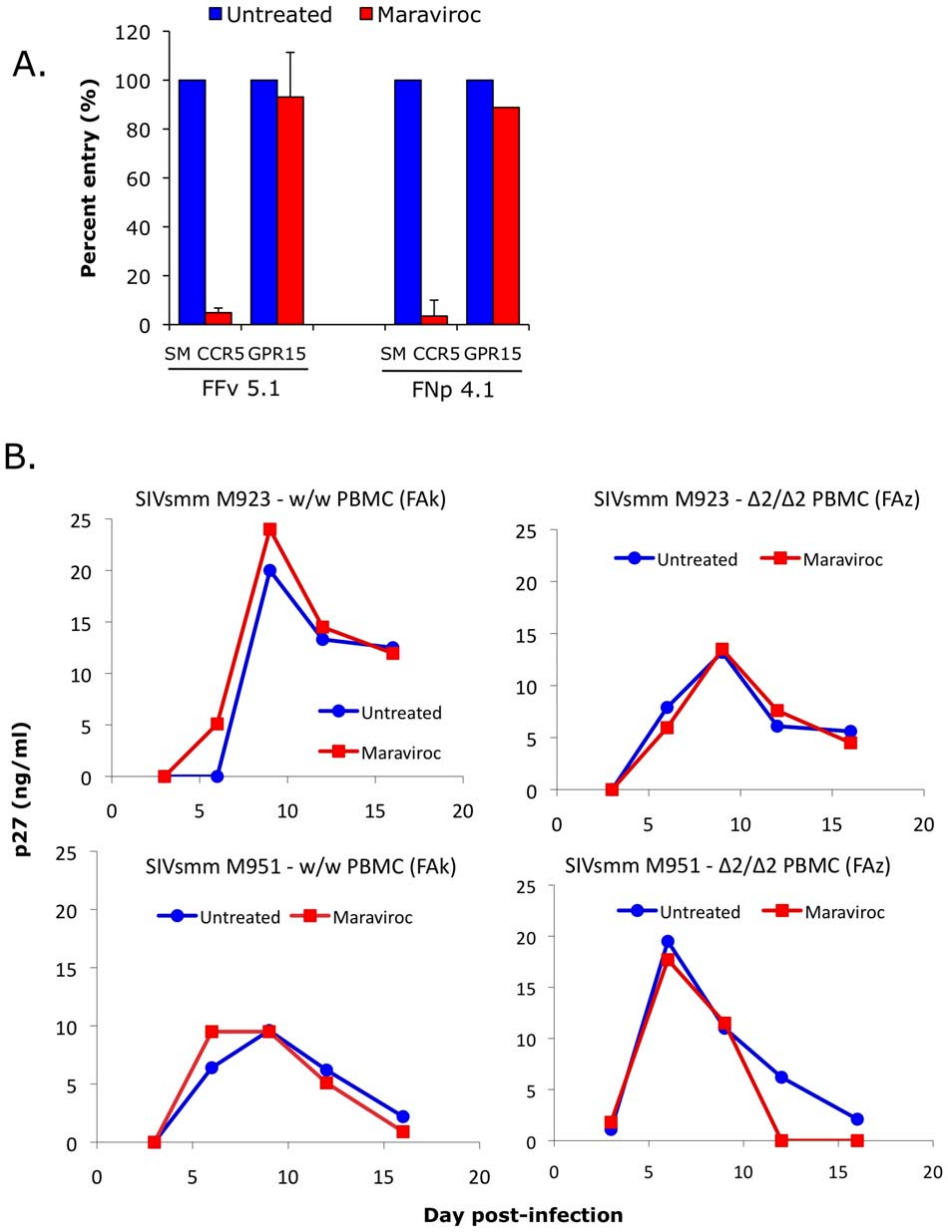
Effect of CCR5 blocking on SIVsmm use of CCR5 and entry into primary SM PBMC. (A) 293T cells were transfected with CD4 in combination with wild-type smCCR5 or GPR15. Two days post-transfection, target cells were pretreated for one hour with or without the CCR5 antagonist, maraviroc (15uM), and then infected with pseudotype virions carrying SIVsmm Envs from a CCR5 wild-type animal (FFv) and a CCR5-null animal (FNp). Three days later, infection was measured based on RLU (mean ± SD) in cell lysates. (B) Growth curves from infection of primary SM PBMC (FAk: W/W SM; FAz: Δ2/Δ2 SM). Cells were stimulated for 3 days with PHA, then pretreated for one hour with or without maraviroc (15 uM), followed by infection with two different SIVsmm primary isolates (M923 and M951) in the continued presence or absence of maraviroc. SIV Gag p27 antigen levels in viral supernatants were measured by ELISA.

We next investigated the role of non-CCR5 pathways in SIVsmm infection of primary SM cells, utilizing both the specific CCR5 antagonist maraviroc and CCR5-null PBMC derived from CCR5Δ2 homozygous animals. Maraviroc blocks chemokine signaling and HIV-1 Env entry through human and rhesus macaque CCR5 [48], [49], but blocking of SM CCR5 coreceptor function has not been reported. Therefore, we first tested the effect of maraviroc on SIVsmm entry through smCCR5 in transfected cells. As shown in Figure 7A, maraviroc blocked SIVsmm pseudotype infection of target cells expressing CD4 and smCCR5, reducing luciferase expression to the level seen with target cells expressing CD4 alone (data not shown), indicating complete blocking of smCCR5-mediated entry by maraviroc. In contrast, maraviroc did not inhibit SIVsmm entry mediated by GPR15 (Figure 7A), nor did it affect entry by VSV-G pseudotypes (data not shown), confirming that blocking is not a nonspecific effect.

Next we asked if maraviroc would affect productive infection of SM primary PBMC by infectious isolates of SIVsmm. PBMC from an uninfected CCR5 wild-type (FAk) and a CCR5-null (FAz) animals were activated with PHA for three days, and then infected in the presence and absence of maraviroc with two SIVsmm primary isolates (M923 and M951), which were both derived from CCR5 wild-type infected animals. Viral replication was measured as SIV gag p27 levels in supernatants collected periodically post-infection (Figure 7B).

We first noted that both SIVsmm isolates were able to productively infect primary PBMC *in vitro* from the CCR5-null SM (FAz). In these cells, there was no difference in replication associated with CCR5 blocking by maraviroc, as expected given the lack of functional CCR5 encoded by the mutant genes. This result indicates that SIVsmm infection of CCR5-null PBMC can occur independently of CCR5. We then tested CCR5 wild-type PBMC (FAk), and found that SIVsmm established productive infection in both the absence and presence of maraviroc. Furthermore, there was no difference in the level of infection achieved when CCR5 was blocked, based on p27 antigen production. This finding indicates that SIVsmm efficiently enters even CCR5-expressing SM primary PBMC through pathways independent of CCR5.

### CCR5 genotypes in sooty mangabeys with SIV evolution to X4 coreceptor use

Unlike HIV-1 in humans, CXCR4 use by SIV in SM is rare. However, a few exceptions have been noted in which CXCR4 use emerged following experimental infection, which was associated with profound CD4+ T cell loss although not clinical AIDS [47]. Therefore, to ask if restricted CCR5+ target cell availability due to genetic absence of the coreceptor might be linked to CXCR4 emergence, we genotyped two infected CD4-low SM previously described in which CXCR4-using SIVsmm variants emerged [47]. Neither animal possessed a CCR5-null genotype: one was CCR5 homozygous wild type and the other was heterozygous for the CCR5Δ2 allele. Thus, the fact that acquisition of CXCR4 use by SIVsmm can occur but is not associated with animals that genetically lack CCR5 is consistent with the notion that alternative pathway-supported entry *in vivo* is robust and lack of CCR5 does not serve as a driving force in the rare cases with emergence of CXCR4 use.

## Discussion

We unexpectedly found that 8% of sooty mangabeys in a large US captive population lack functional CCR5 due to the high prevalence (29%) of mutations in the CCR5 gene that abrogate cell surface expression and SIV coreceptor function. Despite their CCR5-null status, homozygous mutant animals are susceptible to natural as well as experimental SIVsmm infection and display viral loads only modestly lower than CCR5 wild-type animals. *In vitro*, SIVsmm enters primary SM lymphocytes independently of CCR5, and Envs from both wild type and CCR5-null infected animals use several alternative coreceptors in addition to CCR5, but do not use CXCR4. These data indicate that both CCR5 and alternative coreceptor pathways mediate cell entry and robust viral replication *in vivo*. The recognition that both CCR5-dependent and independent pathways are used in the SM natural host has significant implications for understanding viral tropism *in vivo* and CD4+ T cell subset targeting that may regulate the outcome of natural host infection, and raises important questions about entry coreceptor use in pathogenic non-human primate models of AIDS. This finding also explains the previously obscure reason for widespread and efficient use of alternative coreceptors among the SIVmac/smm family of viruses. Finally, it provides a third example of convergent evolution resulting in disruption of CCR5 function among primates, with consequences for virus-host interactions in SM that differ from both humans homozygous for CCR5Δ32 and red capped mangabeys homozygous for CCR5Δ24.

From the original descriptions of alternative coreceptor use by SIVmac/smm viruses, and subsequently by other SIV strains, the reason for conserved use of these pathways has remained elusive [20], [21], [50]. It has been repeatedly shown that various SIV isolates can infect primary human Δ32 homozygous PBMC independent of both CCR5 and CXCR4, indicating that alternative coreceptors are expressed in a manner that supports infection in primary lymphocytes *ex vivo*, at least in cells of human origin [51], [52], [53], [54], [55], [56]. Our data show for the first time that the alternative coreceptors are used by SIVsmm in primary simian T cells *ex vivo* and, more importantly, *in vivo* in the natural host from which the SIVmac/smm family derived. Several important questions are raised by this finding: (1) are alternative pathways also operative in SM with wild-type CCR5 expression, or are they only relevant if CCR5 is absent; (2) what essential role or selective advantage do they provide SIVsmm in SM infection *in vivo* that has led to conservation of alternative coreceptor entry pathway use; (3) does alternative coreceptor use define a novel population of CCR5-negative target cells that contributes to the ability of host and virus to coexist without disease, and; (4) what role do alternative pathways play in infection of macaques, the nonhuman primate model used to study AIDS.

Prior to this confirmation that alternative pathways are used *in vivo*, it seemed plausible that alternative coreceptor use by SIVsmm/mac was an *in vitro* epiphenomenon of little biological significance. Recognizing that they are operative *in vivo*, it seems more likely that conservation of their use among SIV isolates reflects some role that, if not completely essential, offers a selective advantage for the virus. Comparison of viral load data among the genotype groups showed robust replication in the absence of CCR5, but a step-wise increase associated with the presence of one or two functional CCR5 alleles, and *ex vivo* blocking studies with maraviroc confirmed that both wild-type and CCR5-null primary PBMC possess efficient CCR5-independent entry pathways. On one hand, if the relevant coreceptor(s) are expressed only on cells that also express CCR5, the use of multiple pathways *in vivo* might be an example of functional redundancy acquired by SIV, reminiscent in part of the functional redundancy of the chemokine/chemokine receptor system. On the other hand, if CCR5 and alternative pathways are expressed on distinct or only partially overlapping CD4+ T cell subsets, the 0.5 log10 VL difference between Δ/Δ and W/W animals may also be consistent with separate components of plasma viremia supported by CCR5 and non-CCR5 pathways (and an intermediate gene-dosage effect in the presence of one CCR5 allele). It is notable that the degree of depletion in SM gut CD4+ T cells exceeds the proportion that express detectable CCR5 [24], and it will be important to determine if this is due to infection and targeting of CCR5-negative cells in wild-type animals mediated by other coreceptors.

As to why alternative coreceptor use is conserved among SIVsmm and related strains, it seems unlikely to result from the 8% prevalence of CCR5-null animals in SM, and more likely reflects a unique role that provides an advantage over CCR5 alone in transmission, establishment of reservoirs or other aspects of infection. Sooty mangabeys overall express very low levels of CCR5 on CD4+ T cells, which has been proposed as an evolutionary adaptive response to “protect” critical target cells and minimize pathogenesis [17], [18]. If so, acquisition of alternative coreceptor use by the virus may have reflected a “counter-measure” to maximize replication capacity, although doing so in a manner that still retains the nonpathogenic nature of infection. Thus, the use of alternative entry pathways *in vivo* might enable infection of a novel CCR5-negative target cell population that is more expendable than CCR5+ cells, allowing the virus to replicate efficiently without causing disease in the face of extremely restricted CCR5 expression. It will therefore be important to define the distribution of cells infected in animals with and without functional CCR5.

An important question raised by these SM findings is whether alternative coreceptors are utilized in pathogenic infection of macaques, which is widely used to model human AIDS. While CCR5 clearly plays a principal role as evidenced by substantial albeit variable viral suppression by CCR5 antagonists [57], [58], levels of CD4+ T cell infection in rhesus macaques can also substantially exceed the proportion of cells that express detectable CCR5 [23]. While it is possible that infection of apparently CCR5-negative targets reflects entry mediated by CCR5 at levels below the threshold detectable by FACS, our findings revive the question of whether it may be mediated by additional entry pathways. Few studies have attempted to address alternative coreceptor use *in vivo*. One report showed that mutations that abrogated GPR15 use by SIVmac had little effect on replication or pathogenesis in rhesus macaques [22]. Similarly, when pigtail macaques (*Macaca nemestrina*) were infected with SIVmne (also derived from SIVsmm), serial isolates exhibited decreasing ability to use alternative entry pathways when assayed *in vitro* [51]. On the other hand, in cynomolgus macaques (*Macaca fascicularis*) infected with SIVsmm, animals with progressive disease showed retention or broadening of alternative coreceptor use while those without disease progression showed narrowing of alternative coreceptor use [52]. Thus, it remains to be determined whether alternative pathways support entry in particular subsets of CD4+ T cells in the macaque model *in vivo*. Of note, emergence of CXCR4 use is common in HIV-1 infection of humans but exceedingly infrequent in macaques infected with SIVmac. HIV-1 rarely uses alternative coreceptors efficiently and evolution to CXCR4 use by HIV-1 is believed to result, in part, from loss of CCR5+ target cells in late stage disease. Thus, the availability of efficient alternative entry pathways may be one reason that SIV rarely evolves to use CXCR4.

The SIVsmm Envs examined here use GPR15 and CXCR6 for entry quite efficiently *in vitro*, and use GPR1 somewhat less efficiently. This result is concordant with coreceptor use patterns of multiple other SIV isolates [22], [23], [30]. In human blood cells, CXCR6 is highly expressed on CD4+ and CD8+ memory but not naïve T cells, and on gamma-delta T and to a lesser extent NK cells [59], although others have reported expression by CD4+ naïve T cells as well [60]. Interestingly, CXCR6 expression is regulated by T cell activation in a pattern that is tightly linked with CCR5 expression in response to some stimuli, but markedly different in response to others [59]. GPR15 is also expressed on lymphoid and myeloid cells but with less information about specific distribution patterns [20], [21], [61]. Of note, both CXCR6 and GPR15 are also highly expressed in intestinal tissues [20], [62], raising the question of whether alternative coreceptor use may be involved in mucosal events that play a central role in infection. Thus, CXCR6 and GPR15 are particularly likely candidates for mediating SM infection independent of CCR5, and further studies are required to determine which one or ones are responsible for entry into primary SM PBMC *ex vivo* and infection *in vivo*.

One of the most important priorities in HIV/AIDS research at present is understanding why infected natural hosts remain healthy while rhesus macaques infected with SIV, humans infected with HIV-1 or HIV-2, and chimpanzees infected with SIVcpz develop AIDS. Many features are shared by non-pathogenic natural host and pathogenic non-natural host infection including sustained high level viremia, vigorous immune activation during acute infection, and extensive depletion of gut mucosal CD4+ T cells. In pathogenic infections it is believed that in addition to infection and loss of short-lived T effector and T effector memory cells, damage to long-lived CD4+ Tcm populations that impairs the capacity to maintain immune cell homeostasis is a critical factor in progressive immunodeficiency [26], [27], [63]. CCR5 levels are profoundly lower on SM CD4+ T cells compared with RM and humans, which may restrict the target cells available for infection *in vivo* [17], [18]. More recent findings indicate that CD4+ Tcm in SM have particularly impaired CCR5 expression upon activation, and this corresponds with markedly lower levels of cell-associated infection in Tcm compared with Tem cells in SM, whereas both populations are similarly infected in RM (G.S. and M.P; unpublished observations). Thus, in addition to the role of CCR5, it will be important to define the distribution and use of other coreceptors by SIVsmm in its natural host as well as in pathogenic rhesus macaque infection. Another prominent difference between pathogenic infection and nonpathogenic natural host infection is the presence of sustained high level generalized immune activation in rhesus and humans, whereas acute infection in natural hosts is associated with transient immune activation that rapidly resolves [11], [29], [35], [64], [65], [66]. Sustained immune activation during chronic infection may be an additional factor driving T cell turnover and depletion. A principal mechanism driving chronic immune activation is believed to be translocation of microbial products due to disruption of gut mucosal barrier integrity that occurs early in infection [31]. However, gut mucosal lymphocyte infection and CD4+ T cell depletion occurs in natural host as well as pathogenic host infection [24], [33]. One potentially critical difference is the loss in human and rhesus macaque infection, but preservation in natural hosts, of mucosal CD4 Th17 cells, which play a critical role in gut mucosal immune defense [35], [36], [37], [38]. Why gut Th17 CD4+ T cells are preserved in infected natural hosts but depleted in other hosts remains to be determined, but the critical role of entry coreceptors in determining tropism and cell subset infection *in vivo* suggest that both CCR5 and alternative coreceptor pathways must be defined in order to understand the targeting versus protection of critical CD4+ cell subsets.

In addition to the YNPRC and TNPRC SM colonies, we also found the Δ2 allele in SM in the Tai forest of Cote d'Ivoire, confirming its presence not just in captive but also in wild-living West African animals, albeit at a lower frequency. Sooty mangabeys at YNPRC were derived from multiple sources and their history is not well documented [67], so it is difficult to know for sure from what geographic regions in West Africa these animals descended. Of note, the Δ2 allele was not described in earlier studies that reported the Δ24 allele in SM from West Africa and the YNPRC colony [43], [44]. However, CCR5 alleles in those studies were screened by PCR amplicon size, which could discriminate a 24 bp size difference but is unlikely to distinguish a 2 bp difference between the wild type and Δ2 alleles.

The identification here of SM CCR5Δ2 brings to three the number of primates known to have a high prevalence of defective CCR5 alleles. Interestingly, each of the three examples of populations with homozygous mutant CCR5 individuals shows distinct patterns of host/virus interactions. For HIV-1 in humans, which have the lowest prevalence of defective CCR5 genes (∼1% among Caucasians), CCR5 use is a stringent requirement for establishment of new infections, and its absence in the host provides almost complete protection from infection even though late-stage variants can use CXCR4 and, occasionally, other coreceptors. In RCM, which have the highest prevalence of CCR5-null individuals (≥70%), SIVrcm has adapted to use CCR2b as a coreceptor and lost the ability to use CCR5 [43]. SIVsmm infection in SM, which have an intermediate prevalence of CCR5-null individuals (8% in this population), demonstrate an intermediate relationship, in which alternative coreceptors efficiently mediate both natural and experimental infection in the absence of CCR5, but CCR5 use by the virus is retained, perhaps because both CCR5 and alternative pathways together maximize replication in the majority of animals, while the non-CCR5 pathways are required in the CCR5-null animals.

It is somewhat unexpected that SIVsmm does not use CCR2b, which is the route of entry taken by SIVrcm in the absence of CCR5. SM and RCM are closely related and sometimes considered sub-species within the same species [43], [68]. The presence of the same CCR5Δ24 allele in RCM and SM has been interpreted as indicating an origin prior to separation of these populations, although it is uncertain if the remarkably high frequency CCR5Δ24 in the RCM population reflects selective pressure exerted by an environmental or infectious cause, or founder effect [43]. Another question raised by our results is whether the SM CCR5Δ2 and the SM/RCM CCR5Δ24 emerged independently, resulting in deletions in the same region due to multiple nucleotide repeats enabling recombination, or given the overlapping sites whether Δ2 emerged first and additional events led to Δ24. Since Δ2 is not expressed, it is unlikely that further deletions would lead to any additional selective advantage, and two separate recombination deletion events in the same region of the same gene seem more probable.

What selective pressures might have led to three independent primate CCR5 deletion alleles is uncertain. CCR5Δ32 has been present in the human population for at least 3000 years [69], far longer than HIV-1, and despite considerable speculation on infectious or other pressures, both what factors fueled its emergence and when it occurred remain enigmatic [70]. In contrast, SIV has been endemic in the SM and RCM populations for much longer, although whether its entry predated separation of the populations is uncertain [68]. The CCR5 mutation is currently not essential for protection from SIV-induced pathogenesis, but it is plausible that each of the mutations result from ancestral evolutionary pressure by pathogenic SIV infection. For SM, genetic abrogation of CCR5 expression may have been an additional, complementary response to control pathogenesis along with phenotypic CCR5 downregulation [18]. If so, in both SM and RCM the virus then acquired mechanisms to circumvent the restriction, by expanding coreceptor use for SIVsmm or switching for SIVrcm, yet the hosts then acquired other additional mechanisms to avoid pathogenesis. Alternatively, it may be that all three CCR5 mutations, human, SM and RCM, reflect evolutionary adaptation to some other as-yet unidentified infectious or other environmental factor acting similarly on all three types of primates. Nevertheless, whatever its origin and frequency, the SM CCR5Δ2 mutation here expands the pathways known to support infection in this important natural host model, the identity, distribution and utilization of which must be taken into account in understanding SM infection *in vivo*, and the similarities or differences from RM infection that determine outcome from infection.

## Materials and Methods

### Ethics statement

All animal experimentation was conducted following guidelines established by the Animal Welfare Act and the NIH for housing and care of laboratory animals and performed in accordance with Institutional regulations after review and approval by the Institutional Animal Care and Use Committees (IACUC) at the Yerkes National Primate Research Center (YNPRC) or the Tulane National Primate Research Center (TNPRC). Studies were also reviewed and approved by the University of Pennsylvania IACUC.

### Animals and primary cells

These studies utilized blood cells from animals housed at the YNPRC or TNPRC. Peripheral blood mononuclear cells (PBMC) were isolated from whole blood using standard density gradient separation methods. For genomic analysis, approximately 0.8×10^6^ PBMC were lysed in DNA lysis buffer (100 mM KCl; 0.1% NP40; 20 mM Tris pH 8.4; 0.5 mg/ml proteinase K; 200ul total volume) and used as a template for PCR amplification. For infection studies, cryopreserved PBMC were thawed under standard conditions and maintained at 10^6^ cells/ml in RPMI supplemented with 10% FBS, 1% glutamine and 1% penicillin-streptomycin, stimulated for 3 days with 5 µg/ml of phytohemagglutinin (PHA; MP Biomedical), infected and then maintained in the same media in the presence of IL-2 (50 U/ml; Novartis). Analysis of wild SM genotypes was carried out on purified DNA derived from fecal specimens collected in Cote d'Ivoire, which were previously characterized by mitochondrial DNA sequence analysis to represent 33 distinct individuals [45].

### Cloning SM CCR5 genes

Full-length SM-CCR5 genes were amplified by PCR from SM genomic DNA using high fidelity DNA polymerase (Phusion; Finnzymes) and primers based on conserved 5′ and 3′ regions of published SM CCR5 coding sequences (forward: 5′-ATG GAC TAT CAA GTG TCA AGT CCA ACC-3′; reverse: 5′-TCA CAA GCC AAC AGA TAT TTC CTG CTC C-3′). PCR reactions contained Phusion polymerase (1 unit) in HF buffer, primers (0.5 uM), dNTPs (0.2 mM) and 200 ng of purified genomic DNA as template in a 50 ul reaction volume. Thermocycling conditions: initial denaturation at 98°C for 45 seconds, followed by 20 cycles of 98°C for 10 seconds, 71°C for 30 seconds and 72°C for 90 seconds, with a final extension step of 72°C for 10 minutes. PCR amplicons were column purified (QIAquick PCR Purification kit; Qiagen) and then used in a second PCR reaction employing primers that incorporated a *HindIII* restriction site at the 5′ end of the coding region and a *BamHI* restriction site at the 3′ end of the CCR5 coding sequence (forward: 5′-GCT GCT ATA AGC TTC CAC CAT GGA CTA TCA AG-3′; reverse: 5′-AGC GAG CGG ATC CTC ACA AGC CAA CAG ATA-3′; restriction sites underlined). Thermocycling conditions for the second PCR reaction were: an initial denaturation step at 98°C for 45 seconds, followed by 5 cycles of 98°C for 10 seconds, 71°C for 45 seconds and 72°C for 60 seconds, followed by 15 cycles of 98°C for 10 seconds, 76°C for 45 seconds and 72°C for 60 seconds, and final extension at 72°C for 10 minutes. CCR5 amplicons were cloned into the expression plasmid pcDNA3.1+ (Invitrogen) using *HindIII* and *BamHI*, and screened by restriction analysis followed by sequence confirmation. Sequences of the smCCR5Δ2, smCCR5Δ24 and smCCR5 wild-type genes cloned here have been deposited in Genbank (accession numbers HM246694, HM246695 and HM246693, respectively).

### CCR5 genotyping of SM

A two-step PCR-based genotyping assay was developed that identifies CCR5 wild-type and CCR5Δ2 alleles based on differential primer annealing, using genomic DNA from lysates of SM PBMC. Genomic DNA was subject to PCR amplification using the first round primers described above, to amplify the entire CCR5 coding region, in reactions that contained Platinum Taq polymerase (1 unit; Invitrogen), 1.5 mM MgCl_2_, primers (0.5 uM each), dNTPs (0.2 mM), 1–3 ul DNA lysate and Taq buffer in 25 ul reaction volumes. Thermocycling conditions were: initial denaturation at 98°C for 45 second followed by 10 cycles of 94°C for 20 seconds, 68°C for 45 seconds, 72°C for 60 seconds and final extension at 72°C for 10 minutes. The product of this reaction (2.5 ul) was then used as a template for two separate second-round amplifications, each of which used a common downstream primer but different upstream primers specific for the wild-type and Δ2 alleles, respectively (forward CCR5 wild-type: 5′-ATC ACT TGG GTG GTG GCT-3′; forward CCR5Δ2: 5′-ATC ACT TGG GTG GTG CGT-3′; common downstream: 5′-GGT GTT CAG GAG AAG GAC AAT GTT G-3′). The second round PCR reaction used the same conditions as the first reaction. Products of the wild-type and Δ2 amplification reactions were visualized by 2% agarose gel electrophoresis and ethidium bromide staining, which demonstrated a 325 base pair product following amplification with wild-type or Δ2 primer pairs, or both for heterozygotes (Figure S1A).

Each animal was also screened for the CCR5Δ24 deletion allele with a two-step PCR-based assay. Products of the first round PCR reaction described above, were subjected to nested amplification with inner primers (forward: 5′-GGC TAT CGT CCA TGC TGT GT-3′; reverse: 5′-GAC CAG CCC CAA GAT GAC TA-3′) and thermocycling conditions as follows: initial denaturation at 94°C for 45 seconds, followed by 25 cycles of 94°C for 20 seconds, 59°C for 45 seconds, 72°C for 60 second, and a final extension at 72°C for 10 minutes. Products were visualized by 3% agarose gel electrophoresis and ethidium bromide staining, yielding a 205 base pair product from the Δ24 allele, which was easily distinguishable from the 227–229 bp product from the wild-type and CCR5 Δ2 alleles (Figure S1B).

As a secondary confirmation of genotypes established by PCR screening, direct bulk sequencing was carried out on PCR amplified genomic DNA. Amplification was done using the outer primer set as described above, except that Phusion high fidelity polymerase was used for 30 cycles. Products were column-purified and sequenced. Genotypes were verified by manual inspection to confirm the presence of uniform sequences for homozygous animals, or detection of expected frameshifts resulting in overlapping sequences for heterozygous animals (Figure S1C).

For fecal-derived samples, 0.5 ug of purified DNA (of which only a fraction reflected host-derived DNA) was PCR amplified for 35 cycles using first round outer primers as described above, and then subjected to nested amplification for 30 cycles using the inner primer set described above. Products were then subjected to direct sequence analysis.

### Pseudotype luciferase reporter virus infections and coreceptor use analysis

SIVsmm Env-mediated entry was analyzed using luciferase-expressing reporter viruses pseudotyped with envelope glycoproteins of interest. Pseudotype viruses were generated by co-transfecting 293T cells with a plasmid encoding the NL4-3-based *env*-deleted luciferase-expressing virus backbone (pNL-luc-E^−^R^+^) [71] along with expression plasmids encoding SIVsmm, SIVmac, HIV-1 or VSV-g envelope glycoproteins. Cells were transfected overnight using Fugene (Roche) and washed the next day to remove residual transfection reagent. Supernatants were collected 2 days later, clarified by centrifugation and stored at −80°C until use. Pseudotype viruses were quantified based on HIV-1 Gag p24 antigen ELISA (PerkinElmer) and virion infectivity measured on U87 cells stably expressing CD4 and CCR5. Inocula were then standardized on the basis of infectivity in U87/CD4/CCR5 cells (1×10^6^ relative light units; RLU).

The ability of pseudotype viruses to use different coreceptors was assayed in target 293T cells expressing CD4 and the coreceptor of interest. Target cells were prepared by co-transfection with expression plasmids carrying CD4 and the desired co-receptor (1ug of each plasmid) using Fugene. Cells were re-plated one day post-transfection at 2×10^4^ cells/well in 96-well plates and then infected the following day with pseudotype reporter viruses using equivalent inocula (1×10^6^ RLU) by spin inoculation for 2 hours at 1200G. Three days later cells were lysed (0.5% Triton X-100 in PBS) and infection quantified on the basis of luciferase production in target cells, determined by adding an equal volume of luciferase substrate (Promega) and measuring luciferase activity in RLU with a luminometer.

### SIV envelope clones and infectious viruses

SIV envelopes used in pseudotype infections were generated from plasma of SIVsmm-infected CCR5 wild-type (FFv) and Δ2 homozygous (FNp) SM by single genome amplification (SGA) using methods and protocols previously described and cloned into pcDNA3.1 using Topo TA (Invitrogen) [72], [73]. Infectious SIVsmm strains M923 and M951 were isolated as previously described [74] and stocks were prepared in primary SM PBMC.

### FACS analysis of wild-type and mutant CCR5 surface expression

293T cells were transfected with wild-type or mutant forms of CCR5 using Fugene according to manufacturer's instructions. One day later cells were detached by incubation in PBS containing 2 mM EDTA, washed in FACS buffer (PBS containing 1% FBS and 0.1% sodium azide), and stained with the CCR5 monoclonal antibody, clone 3A9-[APC] (BD Pharmingen) or isotype-matched control. Cells were analyzed using a FACS Caliber flow cytometer (BD Biosciences) and FloJo software (Tree Star, Inc.) to determine CCR5 surface expression.

### SIV infections of primary SM PBMC

PHA-stimulated SM PBMC were plated in 96-well plates at 2.5×10^5^ cells/well, incubated for 1 hour in the presence or absence of the CCR5 antagonist maraviroc (15 uM; Pfizer), and then infected with SIVsmm strains (M923 and M951) by spin inoculation (1200×g for 2 hours) followed by overnight incubation. The next day cells were washed in PBS and maintained in media containing IL-2 (50 U/ml), with or without maraviroc (15 uM). Cell supernatants were collected periodically for 3 weeks and replication measured by SIV Gag p27 antigen in cell supernatant by ELISA (Advanced BioScience Laboratories).

### Analysis of SM virological and immunological parameters

Clinical data on infected and uninfected SM housed at YNPRC has been reported previously for surveys carried out in 2004–5 and 2006–7 and 2008–9 [75]. CD3, CD4 and CCR5 expression on PBMC were analyzed by FACS and plasma viral loads were measured by real-time PCR as described [11], [75]. For purposes of analysis, any undetectable viral loads were set at 75 copies, the lower limit of detection. In cases where animals seroconverted during the period of observation, data from prior to infection was included with uninfected animals, while data from after infection was included with infected animals. If multiple measurements were available for individual animals for any parameters, mean values were utilized.

### Statistical analysis

Virological and immunological data were compared between genotype groups using ANOVA or Kruskal-Wallis test followed by the Dunn's multiple comparison test for multiple groups. The two-sample proportions test and Chi-Square test were used for comparison between independent groups. Statistical tests were performed using Prism 4.0 software and OpenEpi: Open Source Epidemiologic Statistics for Public Health, Version 2.3. Data were considered significant when P-value was below 0.05.

## Supporting Information

Figure S1 Analysis of sooty mangabey CCR5 genotypes. (A) Genomic DNA was analyzed by PCR in two separate reactions that contained primers specific for the wild-type or Δ2 CCR5 alleles. (B) Genomic DNA was amplified with CCR5-specific primers that generate an amplicon of 227–229 bp for the Δ2 and wild-type alleles, or 205 bp for the Δ24 allele. Note that animals 1–6 in panel A do not correspond to numbers in panel B. (C) Direct sequence validation of genotypes is shown in representative chromatographs of animals from each of the 5 genotypes identified in this analysis: W/W, W/Δ2; Δ2/Δ2; W/Δ24 and Δ2/Δ24. Red arrow indicates the site of Δ2 or Δ24 frameshifts and blue arrow indicates a single nucleotide polymorphism (T in wild-type; G in Δ2 and Δ24 alleles) that results in a coding change (Leu in wild-type; Val in Δ2 and Δ24 alleles).
(0.81 MB TIF)

Figure S2 Lack of dominant negative effect of mutant CCR5 alleles. 293T cells were transfected with wild-type smCCR5 plasmid (1 ug) along with plasmids encoding CCR5Δ2, CCR5Δ24 or pcDNA3 (1 ug of each plasmid). CCR5 expression was determined by staining with mAb 3A9 and FACS analysis.
(0.27 MB TIF)

Figure S3 Blood CD4+ T cell levels in infected sooty mangabeys between genotype groups. (A) CD4+ T cell counts (cells/ul; mean ± SEM; left) and (B) CD4+ T cell as a percentage of CD3+ cells (mean ± SEM; right) from infected animals in the wild-type (n = 60), heterozygote (n = 49) and homozygous mutant (n = 7) genotype groups. CD4+ T cells are not significantly different between groups (p = ns for absolute cell counts by Kruskal-Wallis test; p = ns for %CD4+ cells by ANOVA).
(0.27 MB TIF)

Figure S4 Relative use of alternative coreceptors compared with CCR5. Representative experiment in which CCR5 and alternative coreceptors were tested in parallel with a subset of SIVsmm Env pseudotype viruses.
(0.18 MB TIF)

Table S1 Punnett square analysis of CCR5 allele frequencies among YNPRC animals.
(0.03 MB DOC)

Table S2 Punnett square analysis of CCR5 allele frequencies among TNPRC animals.
(0.03 MB DOC)
