# Supplementary figures and images for: A Novel CCR5 Mutation Common in Sooty Mangabeys Reveals SIVsmm Infection of CCR5-Null Natural Hosts and Efficient Alternative Coreceptor Use In Vivo
Source: PLoS Pathog. 2010 Aug 26;6(8):e1001064. doi: 10.1371/journal.ppat.1001064 (PMC2928783; doi:10.1371/journal.ppat.1001064)

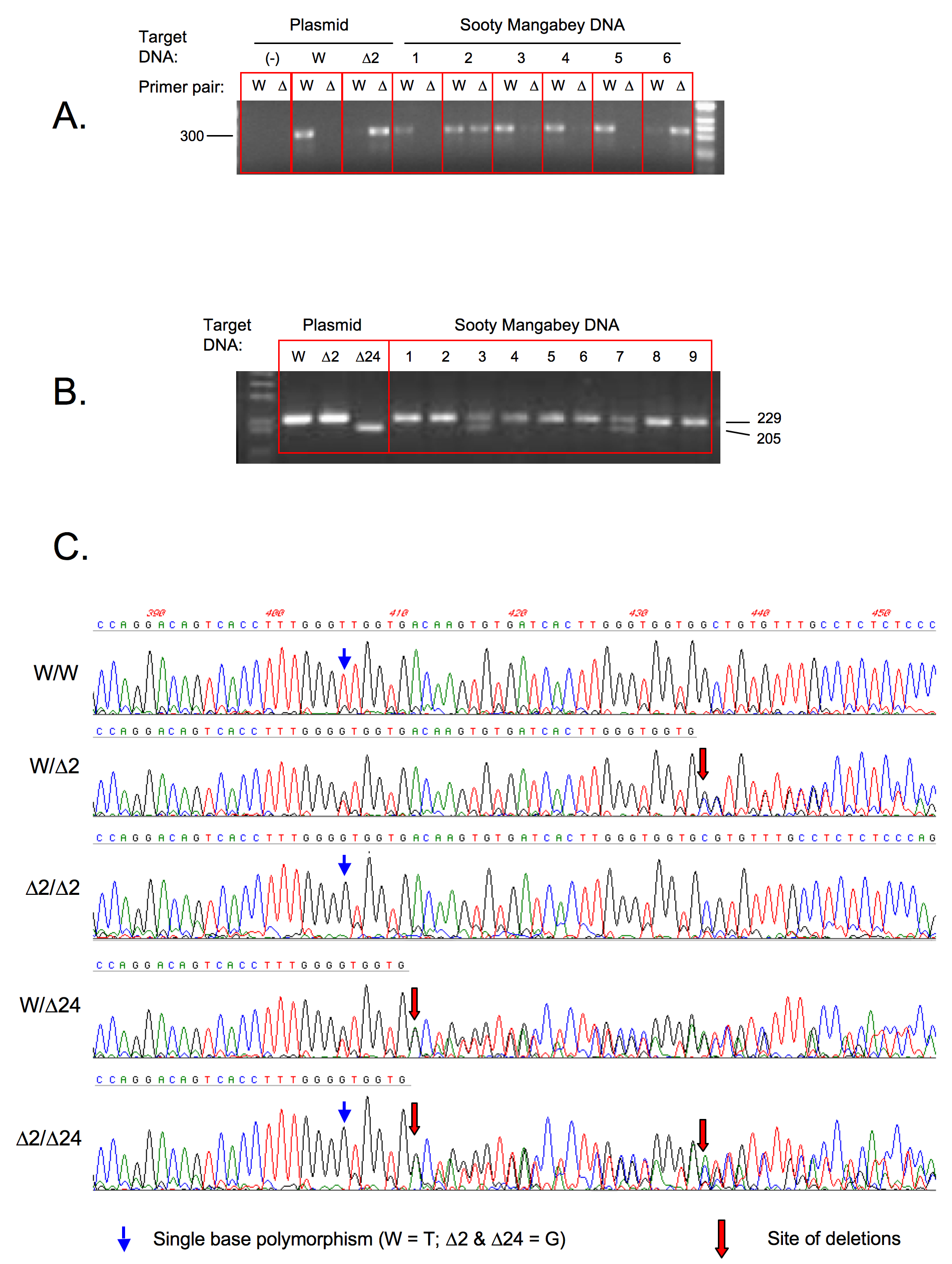

Supplement: Figure S1 — Analysis of sooty mangabey CCR5 genotypes. (A) Genomic DNA was analyzed by PCR in two separate reactions that contained primers specific for the wild-type or Δ2 CCR5 alleles. (B) Genomic DNA was amplified with CCR5-specific primers that generate an amplicon of 227–229 bp for the Δ2 and wild-type alleles, or 205 bp for the Δ24 allele. Note that animals 1–6 in panel A do not correspond to numbers in panel B. (C) Direct sequence validation of genotypes is shown in representative chromatographs of animals from each of the 5 genotypes identified in this analysis: W/W, W/Δ2; Δ2/Δ2; W/Δ24 and Δ2/Δ24. Red arrow indicates the site of Δ2 or Δ24 frameshifts and blue arrow indicates a single nucleotide polymorphism (T in wild-type; G in Δ2 and Δ24 alleles) that results in a coding change (Leu in wild-type; Val in Δ2 and Δ24 alleles). (0.81 MB TIF) [file ppat.1001064.s001.tif]

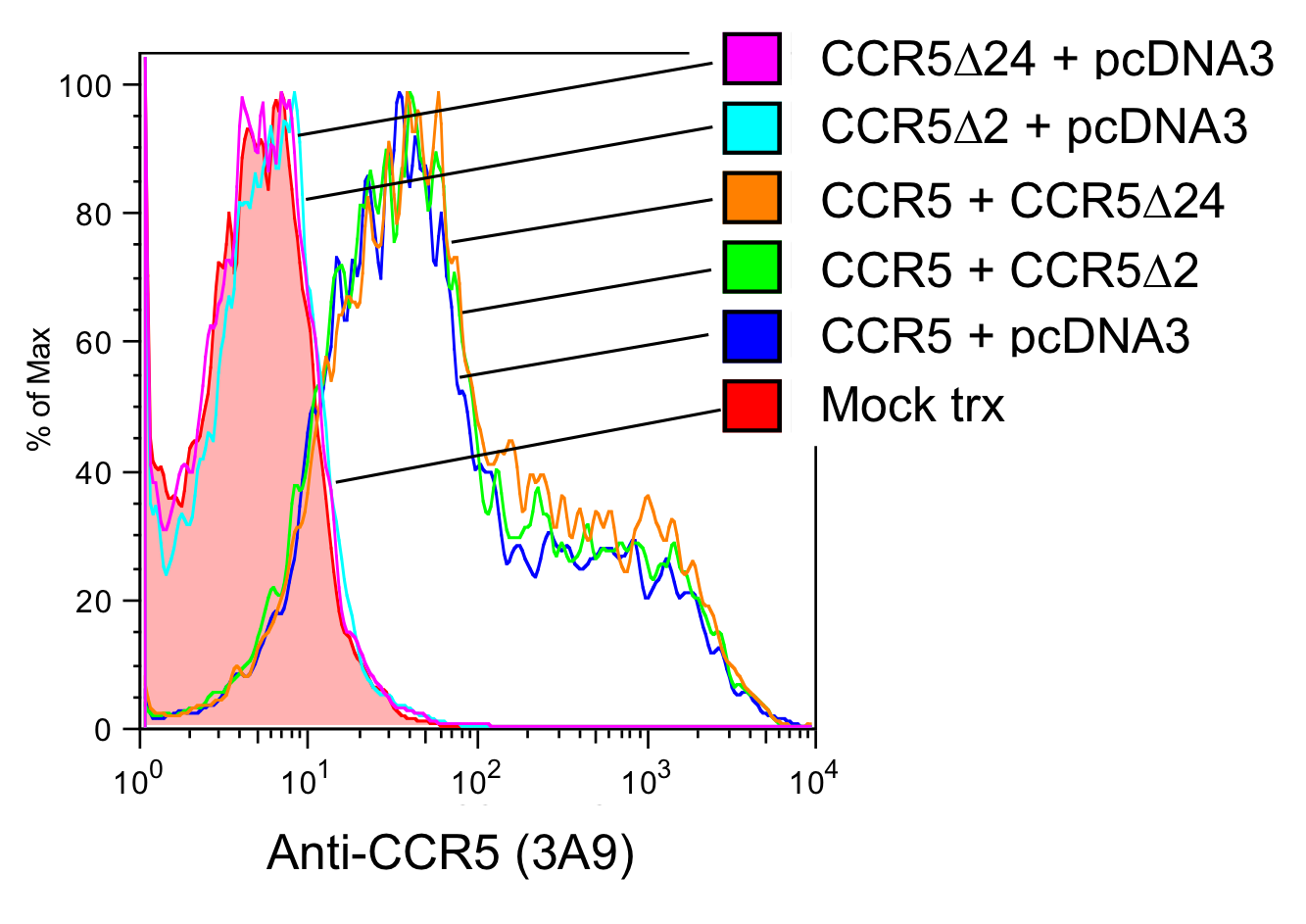

Supplement: Figure S2 — Lack of dominant negative effect of mutant CCR5 alleles. 293T cells were transfected with wild-type smCCR5 plasmid (1 ug) along with plasmids encoding CCR5Δ2, CCR5Δ24 or pcDNA3 (1 ug of each plasmid). CCR5 expression was determined by staining with mAb 3A9 and FACS analysis. (0.27 MB TIF) [file ppat.1001064.s002.tif]

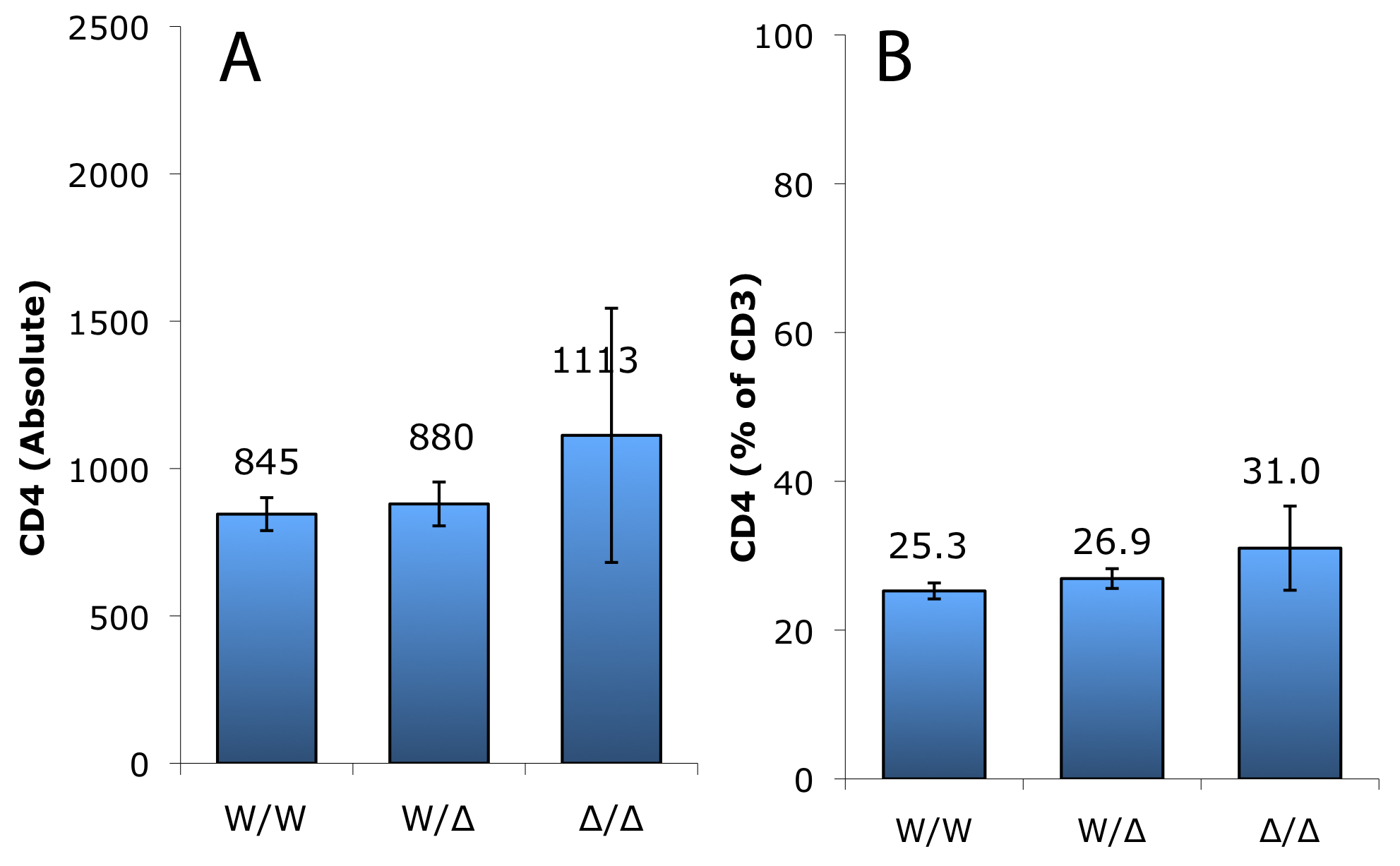

Supplement: Figure S3 — Blood CD4+ T cell levels in infected sooty mangabeys between genotype groups. (A) CD4+ T cell counts (cells/ul; mean ± SEM; left) and (B) CD4+ T cell as a percentage of CD3+ cells (mean ± SEM; right) from infected animals in the wild-type (n = 60), heterozygote (n = 49) and homozygous mutant (n = 7) genotype groups. CD4+ T cells are not significantly different between groups (p = ns for absolute cell counts by Kruskal-Wallis test; p = ns for %CD4+ cells by ANOVA). (0.27 MB TIF) [file ppat.1001064.s003.tif]

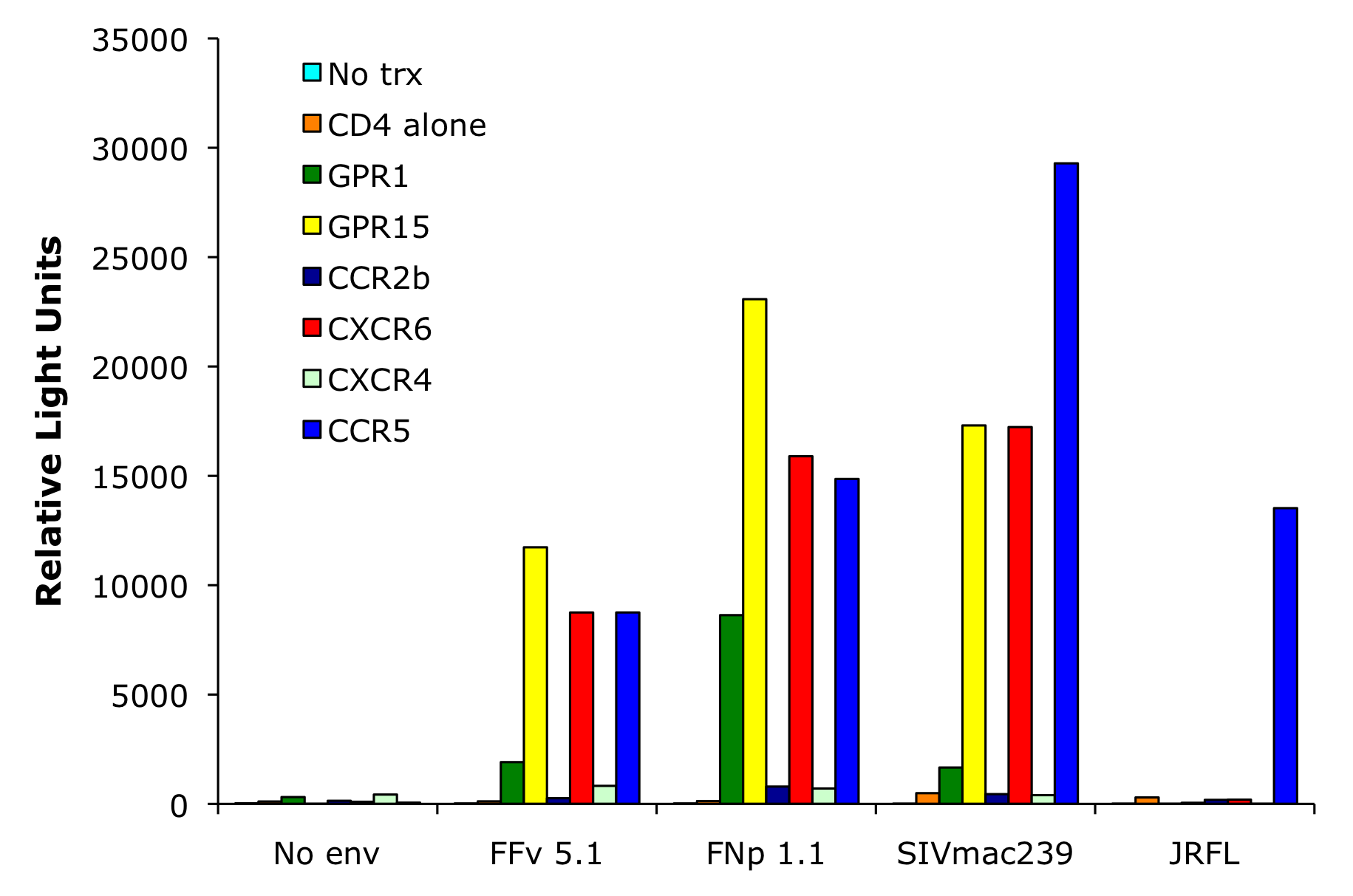

Supplement: Figure S4 — Relative use of alternative coreceptors compared with CCR5. Representative experiment in which CCR5 and alternative coreceptors were tested in parallel with a subset of SIVsmm Env pseudotype viruses. (0.18 MB TIF) [file ppat.1001064.s004.tif]
