# Supplementary material for: A Novel CCR5 Mutation Common in Sooty Mangabeys Reveals SIVsmm Infection of CCR5-Null Natural Hosts and Efficient Alternative Coreceptor Use In Vivo
Source: PLoS Pathog. 2010 Aug 26;6(8):e1001064. doi: 10.1371/journal.ppat.1001064 (PMC2928783; doi:10.1371/journal.ppat.1001064)
Supplement: Table S1 — Punnett square analysis of CCR5 allele frequencies among YNPRC animals. (0.03 MB DOC) [file ppat.1001064.s005.doc]

Table S1. Punnett square analysis of CCR5 allele frequencies among YNPRC animals^1^

| Allelic  Frequency: |  | W  0.71 | ∆2  0.26 | ∆24  0.03 |
| --- | --- | --- | --- | --- |
| W | 0.71 | 0.508 | 0.185 | 0.019 |
| ∆2 | 0.26 | 0.185 | 0.068 | 0.007 |
| ∆24 | 0.03 | 0.019 | 0.007 | 0.001 |

^1^ Allele frequencies were calculated based on genotypes as shown in Table 1.
