# Supplementary material for: A Novel CCR5 Mutation Common in Sooty Mangabeys Reveals SIVsmm Infection of CCR5-Null Natural Hosts and Efficient Alternative Coreceptor Use In Vivo
Source: PLoS Pathog. 2010 Aug 26;6(8):e1001064. doi: 10.1371/journal.ppat.1001064 (PMC2928783; doi:10.1371/journal.ppat.1001064)
Supplement: Table S2 — Punnett square analysis of CCR5 allele frequencies among TNPRC animals. (0.03 MB DOC) [file ppat.1001064.s006.doc]

Table S2. Punnett square analysis of CCR5 allele frequencies among TNPRC animals^1^

| Allelic Frequency: |  | W  0.76 | ∆2  0.19 | ∆24  0.05 |
| --- | --- | --- | --- | --- |
| W | 0.76 | 0.576 | 0.144 | 0.039 |
| ∆2 | 0.19 | 0.144 | 0.036 | 0.010 |
| ∆24 | 0.05 | 0.039 | 0.010 | 0.003 |

^1^ Allele frequencies were calculated based on genotypes as shown in Table 2.
